# Beyond Human Nutrition of Edible Insects: Health Benefits and Safety Aspects

**DOI:** 10.3390/insects13111007

**Published:** 2022-11-01

**Authors:** José E. Aguilar-Toalá, Rosy G. Cruz-Monterrosa, Andrea M. Liceaga

**Affiliations:** 1 Departamento de Ciencias de la Alimentación, División de Ciencias Biológicas y de la Salud, Universidad Autónoma Metropolitana, Unidad Lerma, Av. de las Garzas 10, Col. El Panteón, Lerma de Villada 52005, Estado de México, Mexico; 2 Protein Chemistry and Bioactive Peptides Laboratory, Department of Food Science, Purdue University, 745 Agriculture Mall Dr., West Lafayette, IN 47907, USA

**Keywords:** edible insects, sustainable protein, health benefits, microbial pathogens, allergens, anti-nutritive factors

## Abstract

**Simple Summary:**

Edible insects are a promising alternative food source due to their high nutritional composition and sustainable production. The nutritional value of edible insects is equal or greater than conventional protein sources. Furthermore, it is now known that edible insects can provide health benefits when consumed and if cooked or processed in certain ways, they do not represent safety issues for their consumption.

**Abstract:**

Nowadays, edible insects are considered an outstanding source of nutrients, primarily because they contain high-quality protein, amino acids, and vitamins. Insects are considered a promising alternative protein source towards alleviating future global food shortage problems due to their production considered as being more sustainable by using less agricultural land and water, as well as releasing a smaller amount of greenhouse gas emissions. However, other important aspects to consider about the consumption of edible insects include their health benefits and some safety aspects, which has been relatively overlooked. In this sense, edible insects contain bioactive compounds that can provide diverse bioactivities, such as antioxidant, antihypertensive, anti-inflammatory, antimicrobial, and immunomodulatory with a positive impact on human health. On the other hand, edible insects are a nutrient-rich food that can provide a perfect growth medium for diverse microorganisms, as well as possess some anti-nutritive factors. These two main aspects could represent food safety concerns for consumers. In this context, recent scientific evidence indicates that preservation methods, mainly thermal treatments, utilized in the cooking or processing of edible insects decreased the microbial levels and anti-nutritive factors, which suggests that edible insects do not represent a critical biological risk to humans. Besides, edible insects could have a positive effect on gut microbiota, either by their pre-biotic effect or their antimicrobial activity towards pathogens. Thus, this review is focused on studies related to the health benefits of edible insects and their isolated components, as well as discussion about potential issues related to their microbial content and anti-nutritive factors; this review will provide a synopsis on whether edible insects may be considered safe for human consumption.

## 1. Introduction

**Figure 1:**
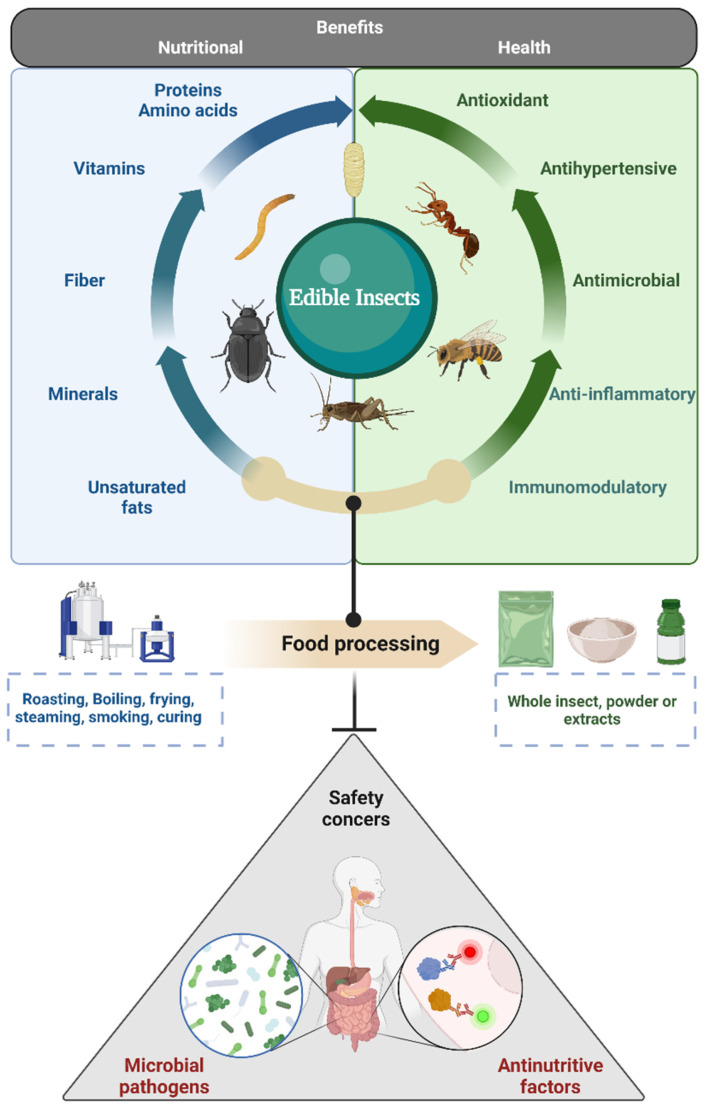
Nutritional and health benefits associated with edible insects. Food processing contributes to the safety of insects for human consumption by targeting safety concerns such as microbial pathogens and anti-nutritive factors. *Created with Biorender.com*.

Insect consumption (i.e., entomophagy) occurs as part of the regular diet of many Asian, African, and Latin American cultures [1,2]. Edible insects are considered an excellent source of nutrients, including high-quality protein, essential amino acids, fiber, mono- and polyunsaturated fats, vitamins and minerals, even at a comparable level of animal protein sources like beef or chicken [2,3,4]. Furthermore, their production is considered to be more sustainable compared to traditional animal protein sources because they require much less water and land use and produce lower greenhouse gas emissions, while possessing a high feed efficiency conversion ratio, high fecundity rates, and short life-cycles [5]. Bearing this in mind, it has been proposed that edible insects can be a promising alternative protein source to alleviate global food shortage problems towards the year 2050, when world food demand is expected to increase by more than 50%; particularly, the demand for animal-based protein is predicted to increase by 70% [6]. In addition to their nutritional and sustainability benefits, insects are reported to be a valuable source of bioactive compounds, such as bioactive peptides [7,8], chitin and chitosan [9,10], phenolic compounds [11,12], and fatty acids [13], providing health benefits (e.g., antioxidant, antihypertensive, anti-inflammatory, antimicrobial, immunomodulatory) when consumed. Thus, edible insects are not only considered a good source of nutrients for human food, but also have the potential to be used as ingredients for nutraceuticals and functional foods (Figure 1).

Despite these nutritional and health benefits, it is important to highlight some aspects related to the potential risk of consumption of edible insects. The above is because, as in the case with other foods, the nutritional composition of edible insects can provide a medium for the growth of pathogenic microorganisms under certain conditions [14]. In addition, it has been reported that some edible insects can present anti-nutritive factors in their composition [15] (Figure 1). On the other hand, in addition to the type of insect, considering that edible insects could be harvested from the wild or from farming facilities, and that they could be processed in different ways (e.g., steaming, roasting, smoking, frying, stewing, and curing, among others) for their consumption [16], it is expected to observe differences in the microbial profile and nutritional composition. This in turn can influence the presence of the anti-nutritive compounds. Overall, these aspects can represent a food safety concern for edible insect consumption and should continue to be a priority for scientists and food industry.

Based on the above, this review explores the potential health benefits of edible insects and their components, as well as important safety aspects related to microbial and anti-nutritive concerns. This review will also discuss some strategies that have been demonstrated to help alleviate these safety concerns, and the current challenges and opportunities that exist in the development of insect-based foods.

## 2. Health Benefits of Edible Insects

Edible insects are reported to contain bioactive compounds that can exhibit a wide spectrum of bioactivities such as antioxidant [17,18], antihypertensive [18,19], anti-inflammatory [7,20], antimicrobial [9,21], and immunomodulatory [22,23] with a positive impact in human health. The bioactivities of various edible insect species have been tested using in vitro assays and in vivo models, either as extracts from the whole insect or as isolated compounds. A summary of recent literature on this topic is depicted in Table 1. 

In the majority of the studies, antioxidant activity was the most evaluated bioactivity, followed by antihypertensive, anti-inflammatory, antimicrobial, and immunomodulatory activities. Likewise, most studies have focused on yellow mealworms (*Tenebrio molitor*) and house crickets (*Acheta domesticus*). This is because these two insect species are among the most promising species for industrial utilization and commercial large-scale production, and are currently approved for human consumption in Europe [31]. 

Edible insects or their components are reported to exhibit antioxidant activity both in vitro and in vivo. For instance, water- and lipo-soluble extracts of various edible insects showed high antioxidant activity. Some samples exhibited two- or even five-fold greater antioxidant capacity than orange juice or olive oil [17]. Similarly, Mudd, Martin-Gonzalez, Ferruzzi and Liceaga [27] reported that bioactive peptides derived from tropical banded crickets (*Gryllodes sigillatus*) had antioxidant activity when evaluated using *Caenorhabditis elegans* as an in vivo model. The results indicated that cricket peptides decreased the reactive oxygen species (ROS) levels and increased the lifespan of *C. elegans* under acute and chronic oxidative stress conditions. In another study using a hypercholesterolemia Wistar rat model [26], the administration of a lipo-soluble extract from silkworms (*Bombyx mori*) increased the antioxidant parameters (e.g., total antioxidant capacity, superoxide dismutase activity, glutathione peroxidase activity) and decreased lipid peroxidation (i.e., malondialdehyde) in both serum and liver tissue. In addition, this extract displayed a hypolipidemic effect by lowering the serum levels of total cholesterol and low-density lipoprotein cholesterol. In contrast, Bergmans et al. [32] included powder of crickets (*Gryllodes sigillatus*) in the diet of malnourished male mice, increasing their body weight and decreasing serum triglycerides.

In other studies, ethanol and ethanol:water extracts from house crickets (*Acheta domesticus*) and yellow mealworms (*Tenebrio molitor*) showed antioxidant activity, which correlated with their total phenolic compounds [28]. Likewise, methanolic extracts from house crickets (*Acheta domesticus*) exhibited antioxidant properties, which were related to their composition of phenolic compounds and proteins [29]. The fermentation of insects and/or insect flours has also shown to produce bioactive metabolites. For example, a study that involved the fermentation of yellow mealworm (*Tenebrio molitor*) and grasshopper (*Sphenarium purpurascens)* flours, reported that the fermented flour samples exhibited antioxidant and antihypertensive activity [30]. 

Other studies have demonstrated that the daily administration of *Tenebrio molitor* larvae powder exhibited an in vivo antiobesity effect in a high fat diet-induced obese mice model through the attenuation of body weight gain and reduction of visceral fat mass [25]. Furthermore, the authors found that the ethanolic extract of this insect showed an anti-adipogenic effect in 3T3-L1 adipocytes by reducing the lipid accumulation and total triglyceride content (up to 90% reduction). Likewise, Nguyen, Kim, Kim, Kim, Kweon, Ji and Koh [26] reported that the administration of silkworm (*Bombyx mori*) larvae powder to a *Drosophila* model increased the healthspan with an extended lifespan compared to flies fed a normal diet. Additionally, the silkworm powder showed a significantly increased resistance to rotenone-induced Parkinson’s disease symptoms as observed by enhanced locomotive control at advanced ages on the fly model. 

Other insect-derived compounds aside from protein, peptides and phenolic compounds have also shown promising biological activity. For instance, chitosan obtained from house cricket (*Acheta domesticus*) and tropical banded cricket (*Gryllodes sigillatus*) chitin, was reported to have hypolipidemic and antimicrobial properties [9]. Both cricket chitosans showed similar values for lipid-binding capacity and were able to inhibit pathogen (*Escherichia coli* ATCC 25,922 and *Listeria innocua* ATCC 33090) growth between 80–100%. Similarly, yellow mealworm (*Tenebrio molitor*) and beetle (*Ulomoides dermestoides*) extracts, rich in saponins, carbohydrates, and proteins, exhibited antimicrobial and antioxidant activities [21]. The antimicrobial activity was tested in Gram-negative (*Proteus vulgaris, Shigella flexnerii*) and Gram-positive (*Bacillus* spp.) strains. Likewise, an aqueous extract from green beetles (*Mimela* sp.) increased its antioxidant activity, enhanced the functions of humoral and cell-mediated immunity, reduced histopathological changes, increased the number of leukocytes, and elevated cytokine levels of TNF-α and IL-6 in immunocompromised mice [24]. Others demonstrated that extracts obtained by the administration of supercritical-fluid CO_2_ from *Tenebrio molitor* larvae powder enhanced cellular and humoral immune response in female Kunming mice [23]. 

These in vitro and in vivo studies have demonstrated that edible insects or their components possess antioxidant, antihypertensive, anti-inflammatory, antimicrobial, and immunomodulatory properties, which suggest that those bioactive compounds can be of use in the enhancement and/or promotion of human health. However, there is a lack of human clinical trials that can confirm their different bioactivities and health benefits in order to support their potential application in the prevention and management of chronic diseases. Therefore, more evidence is needed through well-designed controlled clinical intervention studies along with high-efficiency and resolution techniques to identify and characterize the insect component(s) responsible for these bioactivities. 

## 3. Safety Aspects of Edible Insects

Currently, the safety of edible insects is of great concern for consumers, especially in low-income countries where it is an important component of their culture, gastronomy, and part of their staple diet [1,33]. Many researchers in the food and feed industries have worked to identify and quantify biological and chemical hazards in order to determine if the consumption of edible insects represents a critical risk to humans. The most relevant safety aspects of edible insects are related with their microbiological and anti-nutritive characteristics. However, other safety aspects that should not be overlooked are the presence of pesticides, heavy metals, mycotoxins, and allergens. 

### 3.1. Microbial Safety of Edible Insects

Insects have a complex and diverse community of microorganisms that colonize the insect exoskeleton, mouthparts and gut [34,35]. Some microorganisms protect their insect-host against pathogens, parasitoids, and other parasites [35]. However, like with conventional foods/food ingredients from plants or animals, the consumption of insects entails potential microbiological risks as insects could also serve as vectors for pathogenic microorganisms [1,36]. Particularly, pathogenic bacteria and to a lesser extent viruses, rickettsia, protozoa, fungi, and nematodes [36]. In fact, the Food and Agriculture Organization (FAO) of the United Nations indicated that typical insect pathogens are taxonomically distinct from vertebrate pathogens and can be regarded as harmless to humans, thus should not be seen as potential human pathogens [37]. Nevertheless, the European Food Safety Authority (EFSA) stipulates that the risks associated with insect consumption as a food source for humans could depend on how the insects were reared and processed. The EFSA concludes that edible insects should not pose any additional risks compared to those associated with other conventional foods or food ingredients derived from plants or animals [38]. However, emerging concerns prevail in consumers regarding the microbial safety of edible insect or food ingredients derived from them.

In this context, some studies on live edible insects have been carried out to determine their microbial profile. For example, Stoops et al. [39] determined the microbial counts (log colony-forming unit (CFU)/g) of two fresh edible insect species. The authors reported high values of microbial counts for yellow mealworm larvae (*Tenebrio molitor*) and grasshoppers (*Locusta migratoria migratorioides*), such as total viable aerobic counts (7.7–8.3 log CFU/g and 7.8–8.6 log CFU/g, respectively), *Enterobacteriaceae* (6.8–7.6 log CFU/g and 7.1–7.6 log CFU/g, respectively), lactic acid bacteria (7.0–7.6 log CFU/g and 7.6–8.5 log CFU/g, respectively), and yeasts and molds (5.2–5.7 log CFU/g and 5.0–5.4 log CFU/g, respectively), which are considered higher than values typically permitted for some ready-to-eat foods [40]. Similarly, Klunder et al. [41] reported microbial counts of fresh yellow mealworm larvae (*Tenebrio molitor*), house (*Acheta domesticus*), and African (*Brachytrupus* sp.) crickets. The authors found values of total viable counts and *Enterobacteriaceae* of 6.7–7.7 log CFU/g and 4.2–6.8 log CFU/g, respectively, in all three insects. Likewise, Ali et al. [42] found high microbial counts of total aerobic mesophilic (7.79 log CFU/g), lactic acid bacteria (5.28 log CFU/g), *Escherichia coli* (5.32 log CFU/g), *Salmonella* (5.48 log CFU/g), yeast (5.22 log CFU/g), fecal (8.32 log CFU/g) and total coliforms (7.13 log CFU/g) in fresh edible grasshoppers. These studies showed that fresh, live edible insects showed generally high microbial counts; however, it is worth noting that edible insects are typically cooked or processed prior to their consumption, which could decrease the microbial load. The most common ways used for cooking edible insects are steaming, roasting, smoking, frying, stewing, and braising, which are often applied to improve the palatability of insects [43]. Additionally, edible insects can be processed into powders, extrudates or as dried whole insects [44]. These processing methods typically involve heat treatments or the removal of water (dehydration), which will have an effect on the microbial count. 

In view of the above, several studies have reported the microbial counts of edible insects cooked using different methods. For example, Klunder, Wolkers-Rooijackers, Korpela and Nout [41] found low microbial counts in different cooked (i.e., boiled, roasted or stir-fried) edible insects, including yellow mealworm larvae (*Tenebrio molitor*), house (*Acheta domesticus*), and African (*Brachytrupus* sp.) crickets. These cooked insects had total viable and *Enterobacteriaceae* counts ranging between <1.7–4.8 log CFU/g and <1–2.7 log CFU/g, respectively. The average microbial counts for their fresh counterpart were ca. 7.2 log CFU/g and 5.5 log CFU/g, for total viable and *Enterobacteriaceae* counts, respectively. Interestingly, the authors noticed that some methods were more effective in lowering the microbial count. For example, *Enterobacteriaceae* were eliminated (<1 log CFU/g) in all the insects analyzed when these were cooked by boiling, but this was not true during roasting. Therefore, the effectiveness of the method applied depends on some factors, such as the type, intensity and duration of the heat treatment. Other researchers Ali, Mohamadou, Saidou, Aoudou and Tchiegang [42] found that fried grasshoppers showed low counts of several microbial populations compared with their fresh counterpart [39]. They reported that the frying process considerably reduced the microbial load of *E. coli* (ca. 3.6 log CFU/g reduction), *Salmonella* (ca. 4.5 log CFU/g reduction), fecal (ca. 5.9 log CFU/g reduction) and total (ca. 4.3 log CFU/g reduction) coliforms. Other microorganisms, or indicators of their presence, such as lactic acid bacteria, yeast and sulfite reducing clostridia (indicator for *Clostridium* sp.), were not detected in the fried grasshoppers. In contrast, Mujuru et al. [45] found that the combination of boiling and open-pan roasting methods was the most effective process to decreases the microbial load in Mopani worms (*Gonimbrasia belinak*) compared with other methods (e.g., drum roasting and solar drying). These two processes effectively eliminated coliforms, *E. coli*, *Salmonella* spp., *Staphylococcus aureus*, yeast and molds, but were not effective towards total bacteria counts. In addition, the use of gloves during degutting lowered *E. coli* and *Staphylococcus aureus* counts compared to when bare hands were used. Thus, the practice of hygienic handling of the insects could assist in improving the safety of edible insects. 

Other studies reported the microbial counts of several processed edible insects (e.g., powders, dried). Garofalo et al. [46] analyzed the microbiota of commercial processed edible insects, namely, powdered small crickets, whole dried small crickets (*Acheta domesticus*), whole dried locusts (*Locusta migratoria*), and whole dried yellow mealworm (*Tenebrio molitor*) larvae. They found low counts for total mesophilic aerobes (<2–4.8 log CFU/g), *Enterobacteriaceae* (<2 log CFU/g), lactic acid bacteria (<2–4.5 log CFU/g), *Clostridium perfringens* spores (<2 log CFU/g), yeasts (<2–5.1 log CFU/g) and molds (<2–3.1 log CFU/g) in all of the insects compared with those reported in the literature for fresh edible insects [39,41,42]. In addition, in this study some pathogens such as *Salmonella* spp. And *Listeria monocytogenes* were not detected. Bonaccorsi et al. [47] analyzed the total viable aerobic and *Enterobacteriaceae* counts of dehydrated species of edible insects and scorpions, including wasps (3.8 and <1 log CFU/g), grasshoppers (4.7 and <1 log CFU/g), crickets (2 and <1 log CFU/g), bamboo worms (5.9 and 4.6 log CFU/g), silkworms (7.56 and 1.70 log CFU/g) an insect-mix (5.40 and 3.95 log CFU/g), and scorpions (3.57 and <1 log CFU/g). In general, their results showed values of microbial counts lower than those reported in the literature for fresh edible insects [39,41,42]. Negative microbial counts have been reported for the presence of *Salmonellae*, *Listeria monocytogenes*, *E. coli* and *Staphylococcus aureus* in different cooked and processed edibles insects, such as deep-fried (*Acheta domesticus*, *Locusta migratoria*, and *Omphisa fuscidentalis*), cooked (*Oxya yezoensis*, *Vespula flaviceps*, and *Bombyx mori*), dried (*Acheta domesticus*, *Locusta migatoria*, *Alphitobius diaperinus*, *Tenebrio molitor*, *Bombyx mori*, *Hermetia illucens*, and *Musca domestica*), and powdered (*Hermetia illucens*, *Tenebrio molitor*) [44]. These studies showed that various heat treatments were the most efficient methods for reducing microbial counts. The most commonly used cooking and processing methods reported in the literature for edible insects are shown in Table 2. These are primarily drying, boiling, roasting and frying, which are often applied to improve the taste and palatability of edible insects; while enzyme-technology, freeze-drying, microwave-drying, sonication, pasteurization, and extrusion are some examples of more commercial processing methods used. The most common bacterial pathogens that have been identified from different cooked and processed edible insects belong to the genera *Escherichia*, *Staphylococcus*, *Salmonella*, and *Clostridium*. Most studies on edible insects reported in the literature involve insects that were harvested from the wild and to a lesser extent, reared at farms; however, semi-domestication and indoor farming have increased insect availability and the sustainability of edible insect production [16]. In fact, current food regulations in Europe and the United States require that insects destined for human food should not be collected in the wild and thus must be grown in approved insect rearing facilities [48]. The main reason to recommend the consumption of reared insects rather than those collected in the wild is because the controlled environment in the farm facility makes it possible to regulate and prevent sources of contamination as opposed to the uncontrolled conditions for insects harvested in the wild. Currently, the majority of commercial-scale edible insect farms are located in Europe (e.g., France and Netherlands) and North America (Canada and USA). These farms mainly rear crickets (e.g., *Acheta domesticus*) and yellow mealworms (*Tenebrio molitor*) and rely on their own core breeding stock to ensure a great production of insect biomass, limiting the possibility of introducing diseases into the system [33]. The European Food Safety Authority (EFSA) panel on nutrition (Novel Food and Food Allergens) considered that house crickets (*Acheta domesticus*) reared in closed farming systems are safe for human consumption [49]. The same panel declared that house cricket powder, up to 250 μg/mL, showed no cytotoxic effect on three human cell types (HL60, HeLa, and Caco-2 cells) [49].

Overall, as described above, high counts of microorganisms are present in fresh insects, but with the appropriate thermal treatment, the microbial load can be eliminated. The most common cooking and commercial processing methods reported in the literature for edible insects are also widely used in the food industry for other foodstuffs in order to eliminate spoilage- and disease-causing microorganisms [16,48]. As previously discussed, the effectiveness of the heat treatment depends on factors such as the type, intensity, and duration of the treatment as well as the food composition (e.g., high fat content, pH, water content, etc.). For instance, solar drying can be achieved in 2–5 days at 20–35 °C, which results in a low-temperature and long-time treatment. Other methods use high-temperature and short-time treatments, such as oven drying (8–24 h at 66–80 °C) [16]. Contrary to common foods, important thermal parameters (i.e., thermal death rate curves) for the inactivation of microorganisms in edible insects have not been thoroughly examined [50]. Some of these parameters include the D-value, which indicates the time in minutes required for a 1-log reduction (i.e., kill 90% of the microbial population) at a particular temperature, and the z-value that determines the temperature (°C or °F) required for a specific thermal death curve to pass through one log cycle. Different microorganisms in a given food will have different z-values and thus the z-value indicates the resistance of a microbial population to changing temperature [50,51]. In the future, these important parameters will need to be established for insect-foods and ingredients as they continue to be formulated for human consumption.

**Table 2:** Summary of most common cooking and commercial processing methods reported in the literature for edible insects (adapted from [52]).

Cooking or Processing Method	Insect Species	References
Boiling, blanching, steaming, sautéed	*Alphitobius diaperinus* (beetle) *Ruspolia differens* (grasshopper) *Tenebrio molitor* (yellow mealworm) *Acheta domesticus* (house cricket)	[53,54,55,56,57,58,59]
Drying (sun, lyophilization, fluidized-bed, microwave-assisted)	*Ruspolia nitidula* (grasshopper) *Rhynchophorus phoenicis* (palm weevil) *Ruspolia differens* (longhorn grasshopper) *Tenebrio molitor* (yellow mealworm) *Polyrhachis vicina* Roger (Black ant) *Nauphoeta cinerea* (speckled cockroach)	[53,55,56,60,61,62,63,64,65,66,67]
Enzyme technology; extrusion; sonication, fermentation, ultrasonication, pasteurization, microwave-assisted extractions	*Acheta domesticus* (house cricket) *Schistocerca gregaria* (desert locust) *Spodoptera littoralis* (leaf worm), *Gryllodes sigillatus* (tropical banded cricket) *Tenebrio molitor* (yellow mealworm)	[28,30,68,69,70,71,72,73,74]

In addition to proteins, other insect components including fiber, lipids, vitamins, minerals and phenolic compounds could serve multiple functions in the food and beverage industries. Therefore, their processing and safety also needs to be evaluated. The chitin content in edible insects ranges from 5 to 15% [43]. For example, the chitin content is 6% in giant mealworm larvae, 12% in common mealworm pupa, and 13% in common mealworm larvae [74]. Due to the high chitin content of some edible insect species, they can be a good source of dietary fiber, and chitin has been associated with improving human gut microbiota due to its prebiotic potential. In a 2018 human trial, it was demonstrated that the consumption of chitin-rich cricket powders increased by 5.7-fold the probiotic *Bifidobacterium animalis* present in the gut microbiota of participants [75]. In another study, cricket chitin enhanced the growth of *Lactobacillus rhamnosus* GG and inhibited the growth of *E. coli* TG in in vitro assays [76]. In addition, it has been reported that cricket chitin and chitosan showed antimicrobial properties towards some pathogenic bacteria, such as *Salmonella typhimurium*, *E. coli, Listeria innocua,* and *Vibrio cholera* [9,77]. These properties highlight how edible insects, such as crickets, could have a positive effect on human gut microbiota, either by their prebiotic effect or their antimicrobial activity against common foodborne pathogens. Further in vitro and in vivo studies are necessary to document the prebiotic benefits (or other health benefits) from consumption of insect chitin and chitosan. Additionally, more research is necessary on the antimicrobial properties of chitin derived from edible insect to determine their precise mechanism of action and to validate their functional suitability as ingredients for the development of products with longer shelf life.

### 3.2. Anti-Nutritive Factors of Edible Insects

Anti-nutritive factors are compounds which lower the nutrient utilization or food intake, thereby contributing to impaired gastrointestinal and metabolic performance [78]. In this regard, diverse anti-nutritive factors have been identified in various edible insects. Since the majority of edible insects possess an herbivore feeding behavior, domesticated insects are primarily fed plant diets rich in allelochemicals such as phenolic compounds that although they can be a good source of antioxidants, they can also have anti-nutritive effects [29]. Researchers have determined the presence of four anti-nutrients (i.e., tannin, oxalate, hydrocyanide and phytate) in different insects consumed in Nigeria, such as crickets (*Gymnogryllus lucens*), yam beetles (*Heteroligus meles*), palm weevils (*Rhynchophorus phoenicis*), and grasshoppers (*Zonocerus variegatus*) [15]. Other anti-nutritive factors including cyanogenic glycosides, oxalates, tannins, saponins and alkaloids were identified in *Henicus whellani*, a cricket consumed in south-eastern Zimbabwe [79]. Omotoso [80] found low values of oxalate and phytic acid content, and no presence of tannins, in the larvae of *Cirina forda*. In most cases, these authors found that the level of those anti-nutritive factors are far below the accepted levels established for human consumption. Similarly, heat-resistant thiaminase was detected in the pupae of an African silkworm *Anaphe* spp., which had been reported as the putative cause of a seasonal ataxia and impaired consciousness in Nigerians [81]. In contrast, a study reported that boiling and drying were able to reduce the content of three anti-nutritive factors (i.e., oxalates, tannins, and alkaloids) in the stinkbug *Encosternum delegorguei* typically consumed in Zimbabwe [79]. On the other hand, the EFSA panel on nutrition declared that house cricket (*Acheta domesticus*) powder had concentrations of anti-nutritive factors (e.g., tannins, oxalic acid, phytic acid, hydrogen cyanide, and trypsin inhibitors) comparable to those present in other common foodstuffs [49]; thus, they concluded that the consumption of this insect-based powder was not nutritionally disadvantageous. Other insect compounds such as chitin, can also present challenges as it is speculated to increase the risk of urinary stone formation and chronic responses from insect chitin ingestion have also been reported, but fail to establish a clear correlation between these effects and chitin consumption [82]. 

To best of our knowledge, there are only few studies that have described the presence of anti-nutritive factors in edible insects. Although those studies have reported that these compounds are present in low concentrations or that some processing methods can decrease their content, consequently their presence should not be entirely overlooked. Further research efforts on the extraction protocols and analytical tools are necessary to allow adequate identification of these anti-nutritive factors. Additionally, as the limited evidence suggests, some processing methods (e.g., boiling and drying) could help decrease their concentration, emphasizing the impact of processing methods or supplementary strategies should be further explored and could assist in eliminating these anti-nutritive factors. 

### 3.3. Other Safety Aspects of Edible Insects

Other potential safety aspects of edible insects include the presence of pesticides, heavy metals, mycotoxins, and allergens [83]. 

#### 3.3.1. Pesticides

Different types of chemical pesticides have been detected in edible insects, including insecticides, herbicides, and fungicides [84,85,86]. These pesticides are typically applied to agriculture crops to control pests, such as weeds, fungi, and insects. Edible insects can be contaminated by these pesticides because their direct (i.e., direct spray, irrigation in soil) or indirect (i.e., fed on plants or water that accumulates pesticides) exposure [87,88]. In this context, Poma, Cuykx, Amato, Calaprice, Focant and Covaci [85] performed a screening study to identify what pesticides were present in edible insects (e.g., wax moth—*Galleria mellonella*, migratory locust—*Locusta migratoria*, mealworm beetle—*Tenebrio molitor*, and buffalo worm—*Alphitobius diaperinus*) and insect-based foods (e.g., bugballs made from locust and buffalo worms, cricket croquettes, and bugburgers made from buffalo worms) currently marketed in Belgium. Although the authors did not quantify the pesticide levels, most samples showed the presence of insecticides, such as methoprene, empenthrine, pirimiphos-methyl. Furthermore, some herbicides (e.g., class of chlorbufam, difenzoquate, class morfamquate, tributylphosphate) and fungicides (e.g., azoxystrobine, cycloheximide, tributylphosphate) were detected in samples. However, the levels found (PCBs: 27–2065 pg/g *w/w*; OCPs: 46–368 pg/g *w/w*; BFRs: ≤36 pg/g *w/w*; PFRs: 783–23,800 pg/g *w/w*; dioxin compounds: ≤0.25 pg WHO-TEQ/g *w/w*) were generally lower than those found in traditional animal products. Likewise, De Paepe, Wauters, Van Der Borght, Claes, Huysman, Croubels and Vanhaecke [84] did not find pesticide residues in black soldier flies (*Hermetia illucens*), house crickets (*Acheta domesticus*), or yellow mealworms (*Tenebrio molitor*). However, they did find the presence of the herbicide isoproturon (<1 μg kg^−1^) in grasshopper (*Locusta migratoria*) samples. Brühl, et al. [89] found that flying insect samples collected in different areas in Germany, were contaminated with about 17 different pesticide residues. Although the authors did not identify the insect species analyzed, various herbicides (e.g., metolachlor-S, prosulfocarb, and terbuthylazine) were identified in all the insect samples; the insecticide thiacloprid was also identified in all samples. Similarly, ref. [86] reported that bees (*Apis mellifera*) and their by-products (e.g., fresh stored pollen and beeswax) were contaminated with insecticides (e.g., chlorpyrifos, chlorfenvinphos), and some samples of bees and pollen showed the presence of the pesticides dichlofenthion, carbendazim, and fenitrothion, which are prohibited for their use in the European Union.

It should be noted that in these studies, the presence of pesticides in edible insects did not compromise the health of consumers [84], but their presence should not be overlooked. Just as with conventional animal foods and plant crops, the exposure to pesticides through the consumption of contaminated edible insects could result in long-term health problems, such as cancer, diabetes mellitus, respiratory disorders, neurological disorders, reproductive syndromes, and oxidative stress [90,91]. Even though it is difficult to directly relate the exposure to pesticides and their health problems [92], the severity of these will depend on the pesticide bioaccumulation level, detoxifying system activity, and the antioxidant and immune responses of edible insect consumers [93].

#### 3.3.2. Heavy Metals

Heavy metals can contaminate plants because they absorb metals from the soil and from air pollution. Thus, edible insects can be contaminated by feeding on plants grown in that environment [94,95]. Diverse heavy metals have been identified in edible insects, with the most common being cadmium and lead. For instance, a study in South Africa found high concentrations of cadmium, copper, and manganese in mopane worms (*Imbrasia belina*) [94]. The metal levels were at least three times higher than the European Commission and United States Food and Drug Administration (FDA) standards for the recommended legal limits for human consumption. Similarly, Banjo, et al. [96] found nickel, cadmium, copper, and lead in samples of snout beetles (*Rhynchophocus phoenicis*) and rhinoceros beetles (*Anapleptes trifaciata*). Likewise, Zhang, Lu, Wang and Zheng [95] reported that edible insects consumed in China, such as moths (*Eligma narcissus*), grasshoppers (*Locusta migratoria*), and long-headed grasshoppers (*Acrida chinensis*) showed the presence of mercury, cadmium, and lead. In addition, plant leaf samples from the same sites where these insects were collected were found to have heavy metal levels that were higher in the insects than the plants, indicating that metals bioaccumulated.

On the other hand, edible grasshoppers (*Oxya chinensis formosana*) from Korea exhibited average concentrations of cadmium (0.004 mg/100 mg insect), arsenic (0.12 mg/100 mg insect), lead (0.02 mg/100 mg insect), and mercury (0.0005 mg/100 mg insect), which were below the limits established by the European Commission (Commission Regulation 1881/2006) in foodstuffs [97]. Others also reported the presence of cadmium, arsenic, chromium, and lead in edible insects and insect-based products, which were within the legal limits for human consumption [83]. Similarly, as explained above for pesticides, the toxicological significance of the presence of heavy metals in edible insects should not be disregarded. Exposure to heavy metals has been associated with chronic health problems, including cancer, renal dysfunction, osteoporosis, musculoskeletal diseases, and cardiac failure [98].

#### 3.3.3. Mycotoxins

Mycotoxins, just like microorganisms, are classified as a biological hazard in edible insects. Generally, mycotoxins are produced by phytopathogenic and food spoilage molds belonging to the genera *Fusarium*, *Aspergillus*, and *Penicillium* [99], and are associated with several adverse health effects including cancer and immunosuppression [99,100].

A study by Pradanas-González, et al. [101] reported that insect-based products, namely, cricket (*Acheta domesticus*) flour, silkworm pupae (*Bombyx mori*) powder, and black crickets (*Gryllus bimaculatus*) did not show mycotoxin contamination (e.g., fumonisin B_1_, fumonisin B_2_, T-2 toxin, HT-2 toxin, ochratoxin A, and mycophenolic acid) at quantifiable concentrations. De Paepe, Wauters, Van Der Borght, Claes, Huysman, Croubels and Vanhaecke [84] analyzed the presence of various mycotoxins in various edible insects and found that yellow mealworms (*Tenebrio molitor*) showed a major presence of mycotoxins (e.g., alternariol, HT-2 toxin, and roquefortine), followed by grasshoppers (*Locusta migratoria*) that showed the presence of nicarbazin and nivalenol, while house crickets (*Acheta domesticus*) and black soldier flies (*Hermetia illucens*) were contaminated with alternariol methyl ether and zearalenone, respectively. On the other hand, Musundire, et al. [102] identified a human carcinogen mycotoxin (aflatoxin B_1_) in edible stink bugs (*Encosternum delegorguei*) present at an average concentration of ca. 0.5 ng/g. These insect samples showed levels below the maximum limit recommendations (20 ng/g) established by the World Health Organization (WHO) for that particular mycotoxin. Furthermore, their results showed that the handling and storage conditions influenced the level of aflatoxin contamination because insect samples that were transferred directly into re-used grain bags and woven baskets or were subject to thermal drying showed aflatoxin contamination (0.50 ng/g insect), while those insects transferred directly into clean zip lock bags were absent from mycotoxin contamination. 

Most of the studies discussed here utilized a screening/qualitative method for studying these contaminants; therefore, the need to pursue further studies aimed to reveal contaminants levels in edible insects is evident. A research gap remains present on the possible health effects to consumers due to long-term consumption of edible insects contaminated chemical and biological hazards.

#### 3.3.4. Allergens

Similar to other food proteins, insects can induce mediated allergic reactions in sensitive individuals. Thus, the presence of protein allergens remains a key safety concern in edible insects. Major groups of invertebrates, such as insects (e.g., mites, cockroaches, and crickets), crustaceans (e.g., shrimp, crab, and lobster), and mollusks (e.g., squids, oysters, octopus, and mussels) have been extensively investigated due to their capacity to trigger contact or food allergies in susceptible individuals. Allergic food reactions (post consumption) occur when the immune system overreacts to food substances (i.e., allergens) that induce the production of antibodies (IgE) as an immune defense response. Gier et al. [103] provided a compilation of case studies describing allergic reactions following insect consumption, including allergic reactions following the consumption of beetles, moth larvae, caterpillars, locusts, grasshoppers, cicadas, bees, and the food coloring additive, carmine, derived from cochineal (*Dactylopius coccus*) extract. Reported symptoms ranged from mild localized reactions to severe systemic reactions, such as anaphylactic shock. 

Several studies on different insect species have identified the antigens and IgE-binding proteins responsible for allergic reactions upon exposure and/or consumption [104,105,106]. In insects, tropomyosin, arginine kinase, and glyceraldehyde 3-phosphate dehydrogenase have been identified as highly allergenic, with tropomyosin listed as the major reactive allergen. The protein tropomyosin is known to be a cross-sensitizing allergen in several edible insects due to its reported immunological relationship between crustaceans and insects [107,108,109,110]. Studies have shown immunoreactions associated with teak caterpillar cocoons (*Hyblaea puera*) [111], house crickets (*Acheta domesticus*) [110], silkworm (*Bombyx mori*) [112], grasshoppers [113,114], and tropical banded crickets (*Gryllodes sigillatus*) [18] and a high degree of cross-reactivity between homologous proteins found in crustaceans (shellfish) and other arthropods [108,115,116]. These observations have led to food-label warnings indicating that individuals with a shellfish allergy should avoid eating insects or foods formulated with insect flours/ingredients [117]. A study by Hall and Liceaga [109] demonstrated the shared sequence homology (>60% identity) for cricket tropomyosin and allergens from various species of shellfish, insects, and nematodes. Using immunoinformatics, the authors observed top matches for Lep s 1 silverfish (*Lepisma saccharina*) and Pan b 1.0101 northern shrimp (*Pandalus borealis*) tropomyosin.

Food proteins involved in allergic reactions can be susceptible to physicochemical changes that occur during food processing. For example, a heat treatment applied during food processing can alter the immunochemical reactivity of antigens by modifying the three-dimensional structure of the protein. The protein’s secondary and tertiary structures can be altered as a result of thermal and non-thermal processing, as well as interfacial adsorptions such as air-water (foams), and oil-water (emulsion) systems. Depending on the IgE-binding epitopes, processing can result in protein unfolding and aggregation, including chemical modifications such as glycation [118,119,120]. Nevertheless, preliminary research on the effects of processing technologies on insect allergens seems to indicate that thermal processing methods such as blanching, baking, and frying fail to eliminate mealworm (*Tenebrio molitor*) allergenicity [121]. Thermal processing on locusts (*Patanga succincta*) exhibited different IgE-binding proteins using pooled sera from shrimp-allergic patient sera (n = 16) [122]. Arginine kinase, enolase, and HEX1B were identified as raw locust allergens, whereas fried locust only contained the presence HEX1B and enolase. Broekhoven et al. [123] observed a lower allergic response of tropomyosin IgE cross-reactivity in three edible mealworm species after heat processing and in vitro digestion. 

In contrast, fermentation and enzyme technologies are the main processes known to effectively produce hypo-allergenic foods. During fermentation or the use of enzyme technology, allergen epitope regions are widely exposed to proteases, thus lowering IgE and IgG reactivity [18]. These technologies have been widely used by the food industry for producing hypo-allergenic foods, such as infant formulas [124,125]. Further studies involving other processing technologies, such as high-pressure, microwave heating, ultrasonication, etc., are needed to evaluate their role in lower allergic reactions from insect protein.

## 4. Conclusions and Future Directions

Global food demand is estimated to increase by more than 50% by the year 2050, making it a challenge for agriculture systems across the globe. The negative environmental impact associated with livestock farming to produce conventional meat and dairy products, has led to the proposed consumption of farmed, edible insects in order to ameliorate the food crisis [5]. Edible insects are beneficial to human health because they may induce antioxidant, antihypertensive, anti-inflammatory, antimicrobial, and immunomodulatory activities. Despite the beneficial activities demonstrated using in vitro assays and in vivo animal models, further research is necessary to support their clinical effectiveness in humans. Preliminary evidence shows that edible insects could have a positive effect on gut microbiota, either by their pre-biotic effect or their antimicrobial activity against pathogens. Microbial and anti-nutritive analyses of cooked and processed edible insects have demonstrated that the application of an appropriate processing method (or a combination of several methods) decreased the microbial load and anti-nutritive content. 

As with any other food commodities, the quality and safety of insects needs to be considered. Current food processing methods (e.g., heat-treatments) applied during the cooking or processing of edible insects could guarantee their safety. However, novel technologies (e.g., pulsed electric field) need to be considered, as well as additional hygienic practices in order to guarantee the microbial safety of edible insects during farm-production, processing, and handling. In this context, it is recommended to consume insects reared at farms where their feed and handling conditions are controlled and defined, rather than those insects harvested in wild. The United States and European regulations prohibit the use of edible insects collected in the wild, which has resulted on several new insect farms developed exclusively for human consumption. It is important to emphasize the need to develop proper legislation to ensure husbandry and inspection standards to govern the future production and consumption of edible insects, provide guarantees to producers, and achieve consumer protection.

Although few studies have described the presence of microbial pathogens and anti-nutritive factors in edible insects, adequate processing technologies can help to keep their presence under control. Some studies have also reported that anti-nutritive compounds are present in low concentrations and/or similar to those present in other conventional foods. Nevertheless, their presence should not be overlooked. Further research related to the identification of these anti-nutritive factors, as well as determining the impact of processing methods for eliminating these factors should be explored. Moving forward, other potential hazards such as allergens, heavy metals, dioxins, and pesticides need also to be evaluated in order to determine their potential risk to consumers. Nevertheless, evidence suggests that the consumption of edible insects not only contributes with high-quality nutrients (protein) to our diet, but also can provide other health benefits, and at the same time do not represent a critical biological and anti-nutritive risk to humans that will be any different than those present in other food commodities like meat, dairy, and plants.

## Figures and Tables

**Table 1:** Recent scientific literature related to the health benefits of different edible insect species.

Insect Species	Sample Used or Bioactive Compound Identified	Type of Study	Bioactivities	Reference
Mealworms (*Tenebrio molitor*), buffalo worms (*Alphitobius diaperinus*), Palm worm larvae (*Rhynchophorus ferrugineus*), Evening cicada (*Tanna japonensis*), Black ants (*Lasius niger*), African caterpillars (*Imbrasia oyemensis*), Silkworm (*Bombyx mori*), Grasshoppers (*Calliptamus italicus*), Crickets (*Acheta domesticus*), Mini crickets (*Acheta domesticus*), Giant water bugs (*Lethocerus indicus*), Scolopendra gigantea (*Scolopendra*)	Water and liposoluble extracts	In vitro	Antioxidant	[17]
Crickets (*Gryllodes sigillatus*)	Bioactive peptides	In vitro	Antioxidant, antihypertensive, antidiabetic, antiglycemic, anti-inflammatory	[7,18]
Weaver ants (*Polyrhachis dives*)	Thirteen non-peptide nitrogen compounds (most were identified as alkaloids)	In vitro	Anti-inflammatory, immunosuppressive, renoprotective	[20]
House cricket (*Acheta domesticus*) and tropical banded cricket (*Gryllodes sigillatus*)	Chitin and chitosan	In vitro	Hypolipidemic, antimicrobial	[9]
Mealworms (*Tenebrio molitor*) and Chinese beetle (*Ulomoides dermestoides*)	Extract with main components of saponins, carbohydrates, and proteins	In vitro	Antioxidant and antimicrobial	[21]
Bee pupae	Polypeptide components	In vitro and in vivo	Immunomodulatory	[22]
Mealworms (*Tenebrio molitor*)	Supercritical fluid CO_2_ extract	In vivo	Immunomodulatory	[23]
Green beetle (*Mimela* sp.)	Aqueous extract	In vivo	Antioxidant and immunomodulatory	[24]
Mealworms (*Tenebrio molitor*)	Ethanol extract	In vitro and in vivo	Anti-adipogenic and antiobesity	[25]
Silkworm (*Bombyx mori*)	Powder	In vivo	Anti-Parkinson activity	[26]
Crickets (*Gryllodes sigillatus*)	Bioactive peptides	In vivo	Antioxidant	[27]
House cricket (*Acheta domesticus*) and mealworms (*Tenebrio molitor*)	Polyphenolic ethanol and ethanol: water extracts	In vitro	Antioxidant and antiobesity	[28]
House cricket (*Acheta domesticus*)	Polyphenolic methanolic extracts	In vitro	Antioxidant	[29]
Mealworm (*Tenebrio molitor*) and grasshopper (*Sphenarium purpurascens*)	Flour fermented with *Lactococcus lactis* strains	In vitro	Antioxidant and antihypertensive	[30]
Silkworm (*Bombyx mori*)	Oil	In vivo	Antioxidant and anti-dyslipidemia	[26]

## Data Availability

Not applicable.
